# Determining *Mhc*-*DRB* profiles in wild populations of three congeneric true lemur species by noninvasive methods

**DOI:** 10.1007/s00251-018-1085-z

**Published:** 2018-10-15

**Authors:** Iris I. de Winter, Tamar Qurkhuli, Nanine de Groot, Annemiek J. M. de Vos-Rouweler, Pim van Hooft, Ignas M. A. Heitkönig, Herbert H. T. Prins, Ronald E. Bontrop, Gaby G. M. Doxiadis

**Affiliations:** 1 Resource Ecology Group, Wageningen University, Wageningen, The Netherlands; 2 Department of Biology, Utrecht University, Utrecht, The Netherlands; 3 Institute of Evolutionary Ecology and Conservation Genomics, University of Ulm, Ulm, Germany; 4 Department of Comparative Genetics and Refinement, Biomedical Primate Research Centre, Rijswijk, The Netherlands; 5 Department of Theoretical Biology and Bioinformatics, University of Utrecht, Utrecht, The Netherlands

**Keywords:** Major histocompatibility complex, *Eulemur*, Genetic diversity, Balancing selection, Polymorphism

## Abstract

The major histocompatibility complex (MHC) is a highly polymorphic and polygenic genomic region that plays a crucial role in immune-related diseases. Given the need for comparative studies on the variability of immunologically important genes among wild populations and species, we investigated the allelic variation of MHC class II *DRB* among three congeneric true lemur species: the red-fronted lemur (*Eulemur rufifrons*), red-bellied lemur (*Eulemur rubriventer*), and black lemur (*Eulemur macaco*)*.* We noninvasively collected hair and faecal samples from these species across different regions in Madagascar. We assessed *DRB* exon 2 polymorphism with a newly developed primer set, amplifying nearly all non-synonymous codons of the antigen-binding sites. We defined 26 *DRB* alleles from 45 individuals (17 alleles from *E. rufifrons* (*N* = 18); 5 from *E. rubriventer* (*N* = 7); and 4 from *E. macaco* (*N* = 20). All detected alleles are novel and show high levels of nucleotide (26.8%) and non-synonymous codon polymorphism (39.4%). In these lemur species, we found neither evidence of a duplication of *DRB* genes nor a sharing of alleles among sympatric groups or allopatric populations of the same species. The non-sharing of alleles may be the result of a geographical separation over a long time span and/or different pathogen selection pressures. We found dN/dS rates > 1 in the functionally important antigen recognition sites, providing evidence for balancing selection. Especially for small and isolated populations, quantifying and monitoring *DRB* variation are recommended to establish successful conservation plans that mitigate the possible loss of immunogenetic diversity in lemurs.

**Electronic supplementary material:**

The online version of this article (10.1007/s00251-018-1085-z) contains supplementary material, which is available to authorized users.

## Introduction

The major histocompatibility complex (MHC) is a highly polymorphic and polygenic genomic region and diversity at this complex is considered an important measure of immunocompetence (Klein 1986; Kelley et al. 2005; Piertney and Oliver 2006). The MHC class I and II genes play a crucial role in innate and adaptive immunity, having a major impact on disease resistance (Oliver and Piertney 2006; Schwensow et al. 2007; Rioux et al. 2009; Savage and Zamudio 2011). These genes encode cell surface receptors that present antigens derived from intra- and extracellular parasites and pathogens to T lymphocytes that may consequently initiate an immune response (Germain 1994; Rammensee et al. 1995). Therefore, the MHC genotype determines the diversity of parasites and pathogens that can be recognised, and correlations between particular MHC alleles, high allelic diversity, and number of MHC genes on the one hand and disease resistance on the other have been demonstrated across vertebrate taxa (Briles et al. 1983; Langefors et al. 2001; Schad et al. 2005). For this reason, genetic variation in functionally important MHC gene families plays a central role in vertebrate immunity and in the viability and long-term survival of wildlife populations (Piertney and Oliver 2006; Siddle et al. 2007; Radwan et al. 2010).

The MHC class II region varies between species in the number and presence of genes (Kelley et al. 2005). Some MHC gene families are highly variable, not only in number of alleles but also in the extent of sequence variation between alleles. Variation in the MHC is generated at multiple levels. Animals interact with their immediate environment and are exposed continuously to parasites and pathogens. Selection processes increase resistance to such pressures by generating allelic variation through mutation and recombination, which is well reflected in the diversity patterns of MHC genes (Penn et al. 2002; Kurtz et al. 2006). MHC polymorphism can be similar among species due to their co-ancestry (Klein 1987; Figueroa et al. 1988; McConnell et al. 1988).

The high allelic variation is maintained by balancing selection (Sommer 2005; Piertney and Oliver 2006; Spurgin and Richardson 2010; Grogan et al. 2016), with an increased ratio of non-synonymous over synonymous substitutions at the functionally important antigen-binding sites (ABS) (Garrigan and Hedrick 2003; Fijarczyk and Babik 2015). Balancing selection can be attributed to two processes. First, it can occur when heterozygous individuals are favoured, likely as a result of their ability to respond to a broader array of pathogens than homozygotes (Doherty and Zinkernagel 1975). Second, frequency-dependent selection assumes a co-evolutionary arms race between hosts and pathogens (Takahata and Nei 1990). Polymorphism is maintained when rare alleles are more resistant to pathogens, and are consequently favoured and spread through the population. As soon as parasites have developed antigenicity for these antigens, new, rare alleles will have selective advantage and lead to genetic diversity in populations (Takahata and Nei 1990; Borghans et al. 2004).

Gene duplications and deletions can occur in MHC regions in many primate species (Klein et al. 1993; Nei et al. 1997; Kulski et al. 1999), which result in copy number variation (CNV) among individuals (Slierendregt et al. 1994; Kulski et al. 1999; Doxiadis et al. 2010) and between species (Adams and Parham 2001; Kelley et al. 2005). However, selection against deleterious gene duplications in the MHC operates as well (Shiina et al. 2006), and CNV can also be lost due to genetic drift (Schrider and Hahn 2010; Eimes et al. 2011).

For decades, much has been known about MHC variation, structure, and evolution in humans, captive non-human primates, and other model organisms (Klein 1986; Root-Bernstein 2005). Most of these previous studies focused on the second exon of the MHC II *DRB* gene(s), because this exon encodes functionally important peptides of the antigen-binding site (ABS) (Harf and Sommer 2005). Since exon 2 has been described to be the most polymorphic part in many class II genes, it is therefore assumed to be involved in the susceptibility to specific pathogens (Brown et al. 1993). The class II genes are physically linked, and alleles on these genes are in strong linkage disequilibrium (Marsh et al. 1999). Therefore, *DRB* gene diversity patterns can be a good indicator for the genetic variation in other class II genes, and even for other less closely linked MHC genes (Kelley et al. 2005). In addition, the MHC system is one of the few genetic systems where balancing selection has been revealed in humans and rodents under laboratory conditions and where studies on various captive or semi-captive breeding primate populations exist (Lafont et al. 2007; Schwensow et al. 2007), although comparatively little research has been done on wild populations of mammals, and on primates in particular (e.g., Tung et al. 2015). Captive populations usually guarantee an easy access to blood or other tissue types, which results in the high quality and quantity of DNA extracts. In contrast, studies on the variation in the MHC system of free-ranging wild animal populations, including lemurs, are still rare compared to those on captive animals (Bernatchez and Landry 2003; Kaesler et al. 2017). For animal welfare or technical reasons, such studies often rely on noninvasive sampling for genetic and molecular ecology research. This involves challenges to error-free genotyping from a low quantity of low-quality materials.

Over the past century, lemurs have experienced major declines in range size, and nearly half of all lemur species in Madagascar are threatened with extinction as a result of anthropogenic habitat disturbance and unsustainable hunting (Mittermeier et al. 2010). This study focuses on three species of true lemurs, genus *Eulemur*, family Lemuridae: the red-fronted lemur (*Eulemur rufifrons*), the red-bellied lemur (*Eulemur rubriventer*), and the black lemur (*Eulemur macaco*), each of which diverged about 4.5 million years ago (mya) (Yoder 2007; Markolf et al. 2013). Two of these species (*E*. *rufifrons* and *E*. *rubriventer*) live sympatrically but do not hybridise, and all other *Eulemur* populations are geographically isolated (Markolf et al. 2013). Owing to their highly ecological flexibility, different species of the genus *Eulemur* occupy most biogeographic regions of Madagascar (Johnson 2006), including some of the smaller peripheral islands (Colquhoun 1993). At the same time, they show many similarities in morphology and physiology, as they are genetically closely related (Markolf et al. 2013). Therefore, this genus provides an opportunity to assess the importance of environmental differences in MHC variation as well as the role of balancing selection, which needs to be clarified in *Eulemurs*.

The specific objective of this study was to investigate the allelic variation of the *DRB* gene of the three species of wild true lemurs in different congeneric species across the island Madagascar. In this study, we used a new set of primers that amplify elongated fragments including amino acids 9–13 of exon 2, which represent one of the most important antigen-binding motifs of the beta chain of *DRB*. As a result, this study provides a baseline from which to expand further exploration of lemur MHC in conjunction with wildlife diseases, demographic processes, and other selective forces.

## Materials and methods

### Study species

True lemurs (genus *Eulemur*, family Lemuridae) are morphologically much alike and are medium-sized (body and tail length 30–50 cm, 2–4 kg) arboreal primates that occasionally move quadrupedally on the ground. Their diet consists primarily of fruits, flowers, and leaves (Markolf 2013), although they are all capable of adding alternative food sources such as invertebrates to their diet. This study focuses on three *Eulemur* species: the red-fronted lemur (*Eulemur rufifrons*), the red-bellied lemur (*E*. *rubriventer*), and the black lemur (*E*. *macaco*). The main difference between these *Eulemur* species is their social organisation, including group size: *Eulemur rufifrons* and *E*. *macaco* live in multi-male, multi-female groups of 4 to 18 individuals (Overdorff 1996; Erhart and Overdorff 2008), whereas *E*. *rubriventer* lives in small monogamous groups from two to five individuals (Overdorff 1996). All three species are listed in the IUCN Red List of Threatened Species: *Eulemur rufifrons* as ‘near threatened’, and *E*. *rubriventer* and *E*. *macaco* as ‘vulnerable’ (Andriaholinirina et al. 2014).

### Study site

**Fig. 1:**
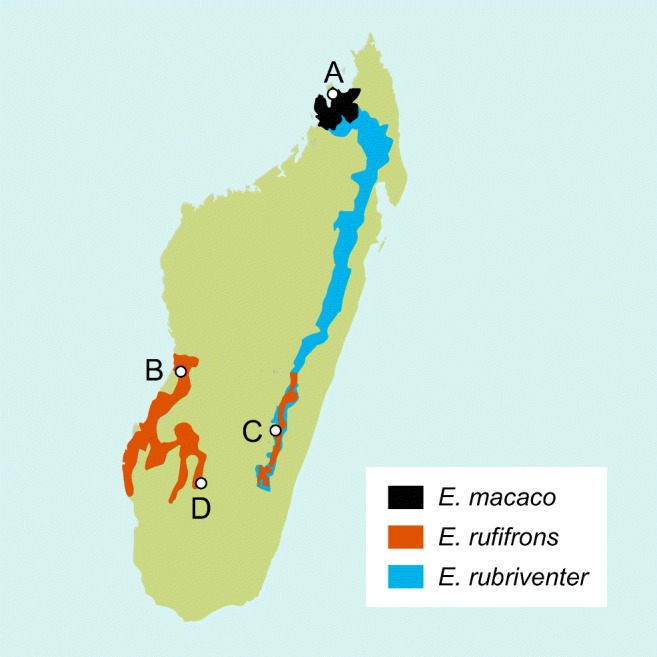
Study sites and geographic ranges of the study species. Map of Madagascar with the geographic ranges of the three study species, *Eulemur macaco*, *E*. *rufifrons*, and *E*. *rubriventer*, and the corresponding sites where samples were collected, (A) Nosy Komba, (B) Kirindy Forest, (C) Ranomafana NP, and (D) Isalo NP

We collected biological materials from three different lemur species in four different field sites across Madagascar. We collected samples from *Eulemur rufifrons* and *E*. *rubriventer* in Ranomafana National Park (NP) (N, − 21.32; E, 47.40), samples from *E*. *rufifrons* in Isalo NP (N, − 22.47; E, 45.26) and Kirindy Forest (N, − 20.07; E, 44.66), and samples from *E*. *macaco* on Nosy Komba (N, − 13.46; E, 48.35) (Fig. 1). Kirindy Forest and Isalo are located on the western side of Madagascar and consist of dry deciduous forest with pronounced seasonality. These western regions have a higher annual mean temperature than the eastern rainforests and receive less rainfall (Goodman et al. 2005). Ranomafana NP is a humid rainforest located on the eastern side of Madagascar. The island Nosy Komba is located in the north-west of Madagascar and is covered with tropical vegetation.

### Sample collection

In a noninvasive manner, we collected samples (*N =* 45 individuals; *N =* 51 faecal; and *N =* 20 hair samples) from individuals between October 2013 and May 2014. Immediately after animals had defecated, fresh faecal samples (3–4 g) of adult lemurs were collected with a pincer that we cleaned with ethanol to avoid contamination. We aimed to sample all adult individuals within a social group and prevented duplication by identifying each individual on the basis of its morphology. From some individuals, both hair and faecal samples were collected. Within 12 h after collection, we stored the collected samples in 15 mL tubes containing 15 g of silica beads to desiccate the faeces (Wasser et al. 1997). We stored these samples in the shade at ambient temperatures until further analyses by colleagues in the Department of Comparative Genetics & Refinement, Biomedical Primate Research Centre (BPRC) in the Netherlands. In addition to faecal samples, hair was collected opportunistically when possible and stored in small ziplock bags. When the lemurs approached closely enough, a tuft of hair was removed from the hip region. Sample collection and export were approved by the trilateral commission (CAFF/CORE) in Madagascar (permits 297/13 and 143/14/MEF/SG/DGF/DCB.SAP/SCBSE).

### DNA extraction

Total DNA was extracted from faeces by using the QIAamp® DNA Stool kit (Qiagen) according to the manufacturer’s guidelines, with a few modifications (Nsubuga et al. 2004), which include the following. At the start of the extraction, (1) we covered the silica beads in 15-ml tubes containing the dried faecal samples with 1.5 to 2 mL of ASL buffer; (2) the tubes were shaken for 12 to 16 h at 25 °C; (3) the supernatant was fully removed from the preservation tubes to extract the DNA; (4) at the end of the extraction, the recommended step of 1-min centrifugation at full speed in a new collection tube was applied; (5) 50 μL of AE buffer was used for elution; and (6) incubation at room temperature for 20 min was followed by centrifugation at full speed for 2 min. DNA from hair was extracted by using the Gentra Puregene tissue kit (Qiagen) according to the manufacturer’s guidelines. DNA quality and quantity were estimated by absorbance at 260/280 nm on the ND-1000 NanoDrop®.

### PCR reaction for DRB exon 2

A 213-bp fragment of *DRB* exon 2 was amplified by PCR using a generic 5′*DRB*-exon 2 primer CGT GTC CCC ACA GCA CGT TTC (Doxiadis et al. 2006) together with the 3′JS2 primer GAT CCC GTA GTT GTG TCT GCA (Schad et al. 2004). The PCR reactions were performed in a 50 μL volume containing 5 units of Platinum *Taq* polymerase (Invitrogen, Paisley, Scotland) with 0.2 μM of each primer, 5 mM MgCl_2_, 0.2 mM of each dNTP, 1X PCR buffer (Invitrogen, Paisley, Scotland), and 50–200 ng DNA. The cycling parameters were a 2-min initial denaturation step at 94 °C, followed by 3 cycles of 90 s at 94 °C, 90 s at 60 °C, and 90 s at 74 °C. This programme was followed by 32 cycles of 30 s at 94 °C, 30 s at 60 °C, and 30 s at 74 °C. A final extension step was performed at 72 °C for 7 min.

### Cloning and sequencing

PCR products were purified using a GeneJet Gel Extraction Kit (Thermo Scientific™), and the purified amplicons were cloned into the pJET vector using the CloneJET PCR cloning kit, both according to the manufacturer’s guidelines (Thermo Scientific™). Next, the cloned amplicons were transformed in *Escherichia coli* XL1-blue cells by using the TransformAid Bacterial Transformation Kit (Thermo Scientific™). Per animal, 24 to 48 bacterial clones were picked, and plasmid DNA was isolated using a standard mini-preparation procedure. The purified plasmid DNA was sequenced on the ABI 3500 genetic analyser (Applied Biosystems, Foster City, USA). The sequencing reaction was performed by using 2 μM pJET primer, 1 μL BigDye terminator, and 2 μL of 5 × sequencing buffer in a total volume of 10 μL (Thermo Scientific™). The resulting sequences were analysed using the Sequence Navigator programme (Applied Biosystems, Foster City, USA). MHC sequences were revised manually by applying the Lasergene 12 SeqMan Pro Sequence Alignment Editor.

### Allele discovery and nomenclature

All sequences were compared in BLAST at GenBank (National Centre for Biotechnology Information, NCBI) and turned out to be novel and related to *Eulemur DRB* exon 2. Only sequences with an identity higher than 95% to already-published lemur *DRB* alleles were considered to be of lemur origin and were selected for further analysis. Furthermore, only sequences that were detected at least two times, either in two different PCRs of the same sample or in two different animals, were accepted as being true and new alleles. The alleles were named numerically based on general principles used for the IPD-MHC 2.0 database (Maccari et al. 2017). We deposited all alleles in GenBank, and they were given the accession numbers MF682987-MF683012.

### Phylogenetic analysis

We constructed a neighbour-joining phylogenetic tree to show phylogenetic relationships among *DRB* alleles of *E*. *rufifrons*, *E*. *rubriventer*, and *E*. *macaco*, with evolutionary distances computed according to the Kimura-2-parameter method (Saitou and Nei 1987). We used a bootstrap consensus tree inferred from 2000 replicates and included both transitions and transversions, assuming rates among sites to have a gamma distribution, with a gamma parameter set to 1. Branches corresponding to partitions reproduced in less than 50% bootstrap replicates were collapsed. The percentage of replicate trees in which the associated taxa clustered together in the bootstrap test is shown. The tree is drawn to scale, with branch lengths in the same units as those of the evolutionary distances used to infer the phylogenetic tree. The analysis involved 26 nucleotide sequences. All positions containing gaps and missing data were eliminated. There was a total of 213 positions in the final dataset. Evolutionary analyses were conducted in MEGA V.5 (Tamura et al. 2011).

### dN/dS calculations

We calculated the relative rate of non-synonymous (dN) and synonymous (dS) substitutions (Nei and Gojobori 1986) with the Jukes-Cantor correction (Jukes et al. 1969) for multiple hits in MEGA. Substitution rates were calculated for the overall sequences and then separately for ABS and non-ABS. Concordance with ABS in human MHC molecules was assumed, with the following beta chain residues: 9, 11, 13, 28, 30, 32, 37, 38, 47, 56, 60, 61, 65, 68, 70, 71, 74, and 78. Therefore, six other residues (i.e., 81, 82, 85, 86, 88, 89) that were identified as ABS (Brown et al. 1993) are not part of the amplicons derived in this study. Statistical differences in dN/dS rates were tested with a Z-test. For all calculations, the alpha level was set at 0.05.

## Results

### DRB allele definition and variation

A total of 26 *DRB* alleles could be identified among 45 individuals of *E*. *rufifrons* (*N =* 18), *E*. *rubriventer* (*N =* 7), and *E*. *macaco* (*N =* 20, Tables 1, 2). *Eulemur rufifrons* showed the highest allelic variation, with 17 different *DRB* alleles (*Eufr*-DRB*01–*17*) defined in 18 animals. Most individuals of this species lived in Kirindy Forest (Table 2, Fig. 1), and, accordingly, most alleles are defined in these animals (Table 1). In three animals from Isalo NP, three other *DRB* alleles were determined along with another two alleles in the two individuals from Ranomafana NP. *Eulemur rubriventer* showed the second highest allelic variation, with five alleles (*Euru*-DRB*01–*05*) among eight animals, whereas *E*. *macaco* was least polymorphic for its *DRB* gene, with only four alleles (*Euma*-DRB*01–*17*) defined in the 20 individuals genotyped (Tables 1, 2).

**Table 1:** All detected alleles (*N =* 26) and the specific individual samples

A. *Eulemur rufifrons*, Kirindy Forest		
#	Allele	Animal ID
1	*Eufr*-DRB*01	K1RF, K5RF
2	*Eufr*-DRB*02	K7RF, K2RF
3	*Eufr*-DRB*03	K10RF
4	*Eufr*-DRB*04	K1RF, K25RF, K4RF, K9RF
5	*Eufr*-DRB*05	K25RF
6	*Eufr*-DRB*06	K6RF
7	*Eufr*-DRB*07	K27RF, K8RF, K5RF
8	*Eufr*-DRB*08	K10RF
9	*Eufr*-DRB*09	K7RF, K2RF
10	*Eufr*-DRB*10	K11RF, K4RF, K3RF
11	*Eufr*-DRB*11	K8RF
12	*Eufr*-DRB*12	K3RF, K11RF
B. *Eulemur rufifrons*, Isalo NP		
13	*Eufr*-DRB*13	I7RF, I4RF
14	*Eufr*-DRB*14	I2RF
15	*Eufr*-DRB*15	I4RF, I7RF, I2RF
C. *Eulemur rufifrons*, Ranomafana NP		
16	*Eufr*-DRB*16	R90RF
17	*Eufr*-DRB*17	R105RF, R90RF
D. *Eulemur rubriventer*, Ranomafana NP		
#	Allele	Animal ID
1	*Euru*-DRB*01	R43RB, R29RB, R27RB
2	*Euru*-DRB*02	R30RB, R27RB
3	*Euru*-DRB*03	R33RB, R29RB, R35RB
4	*Euru*-DRB*04	R30RB
5	*Euru*-DRB*05	R33RB, R34RB, R55RB, R35RB
E. *Eulemur macaco*, Nosy Komba		
#	Allele	Animal ID
1	*Euma*-DRB*01	NK7, NK13, NK19, NK9, NK14, NK2
2	*Euma*-DRB*02	NK21, NK16, NK7, NK17, NK9 NK3, NK1EMA, NK4, NK12, NK1
3	*Euma*-DRB*03	NB1EMA, NK3, NK4, NK16, NK22, NK2, NK12
4	*Euma*-DRB*04	NK19

**Table 2:** Individual MHC class II *DRB* exon 2 genotypes for 45 different lemurs

A. *Eulemur rufifrons*, samples from Kirindy Forest, Isalo NP, and Ranomafana NP			
	Kirindy Forest	Allele 1	Allele 2
1	K1RF	*Eufr*-*DRB 01*	*Eufr-DRB 04*
2	K2RF	*Eufr*-*DRB 02*	*Eufr*-*DRB 09*
3	K3RF	*Eufr*-*DRB 10*	*Eufr*-*DRB 12*
4	K4RF	*Eufr*-*DRB 04*	*Eufr*-*DRB 10*
5	K5RF	*Eufr*-*DRB 01*	*Eufr*-*DRB 07*
6	K6RF	*Eufr*-*DRB 06*	
7	K7RF	*Eufr*-*DRB 02*	*Eufr*-*DRB 09*
8	K8RF	*Eufr*-*DRB 07*	*Eufr*-*DRB 11*
9	K9RF	*Eufr*-*DRB 04*	
10	K10RF	*Eufr*-*DRB 03*	*Eufr*-*DRB 08*
11	K11RF	*Eufr*-*DRB 10*	*Eufr*-*DRB 12*
12	K25RF	*Eufr*-*DRB 04*	*Eufr*-*DRB 05*
13	K27RF	*Eufr*-*DRB 07*	
	Isalo NP	Allele 1	Allele 2
14	I2RF	*Eufr-DRB 14*	*Eufr*-*DRB 15*
15	I4RF	*Eufr-DRB 13*	*Eufr*-*DRB 15*
16	I7RF	*Eufr-DRB 13*	*Eufr*-*DRB 15*
	Ranomafana NP	Allele 1	Allele 2
17	R105RF	*Eufr-DRB 17*	
18	R90RF	*Eufr-DRB 16*	*Eufr*-*DRB 17*
B. *Eulemur rubriventer*, samples from Ranomafana NP			
	Ranomafana NP	Allele 1	Allele 2
1	R43RB	*Euru*-*DRB 01*	
2	R33RB	*Euru*-*DRB 03*	*Euru*-*DRB 05*
3	R34RB	*Euru*-*DRB 05*	
4	R55RB	*Euru*-*DRB 05*	
5	R29RB	*Euru*-*DRB 01*	*Euru-DRB 03*
6	R30RB	*Euru*-*DRB 02*	*Euru-DRB 04*
7	R27RB	*Euru*-*DRB 01*	*Euru-DRB 02*
C. *Eulemur macaco*, samples from Nosy Komba			
	Nosy Komba	Allele 1	Allele 2
1	NK21	*Euma*-*DRB 02*	
2	NK16	*Euma*-*DRB 02*	*Euma*-*DRB 03*
3	NK7	*Euma*-*DRB 01*	*Euma*-*DRB 02*
4	NK17	*Euma*-*DRB 02*	
5	NK3	*Euma*-*DRB 02*	*Euma*-*DRB 03*
6	NK1EMA	*Euma*-*DRB 02*	
7	NB1EMA	*Euma*-*DRB 03*	
8	NK22	*Euma*-*DRB 03*	
9	NK13	*Euma*-*DRB 01*	
10	NK4	*Euma*-*DRB 02*	*Euma*-*DRB 03*
11	NK12	*Euma*-*DRB 02*	*Euma*-*DRB 03*
12	NK1	*Euma*-*DRB 02*	
13	NK2	*Euma*-*DRB 01*	*Euma*-*DRB 03*
14	NK14	*Euma*-*DRB 01*	
15	NK9	*Euma*-*DRB 01*	*Euma*-*DRB 02*
16	NK10	*Euma*-*DRB 02*	*Euma*-*DRB 03*
17	NK19	*Euma*-*DRB 01*	*Euma*-*DRB 04*
18	NK23	*Euma*-*DRB 02*	*Euma*-*DRB 03*
19	NK5	*Euma*-*DRB 02*	*Euma*-*DRB 03*
20	NK15	*Euma*-*DRB 02*	

*Eufr-DRB* was characterised by high polymorphism, with most alleles being observed in just one or two animals, whereas *Euru*-*DRB* but especially *Euma*-*DRB* alleles were detected in far more animals. Five to ten *E*. *macaco* individuals shared the same allele, with the exception of Euma-DRB*04, which was observed in one animal only (Table 1). Most individuals were heterozygous (Table 2; observed heterozygosity: *Eulemur rufifrons*, 0.78; *E*. *rubriventer*, 0.57; *E*. *macaco*, 0.55). None of the animals showed more than two *DRB* alleles, indicating that we did not detect a sign of *DRB* duplication in these species.

**Fig. 2:**
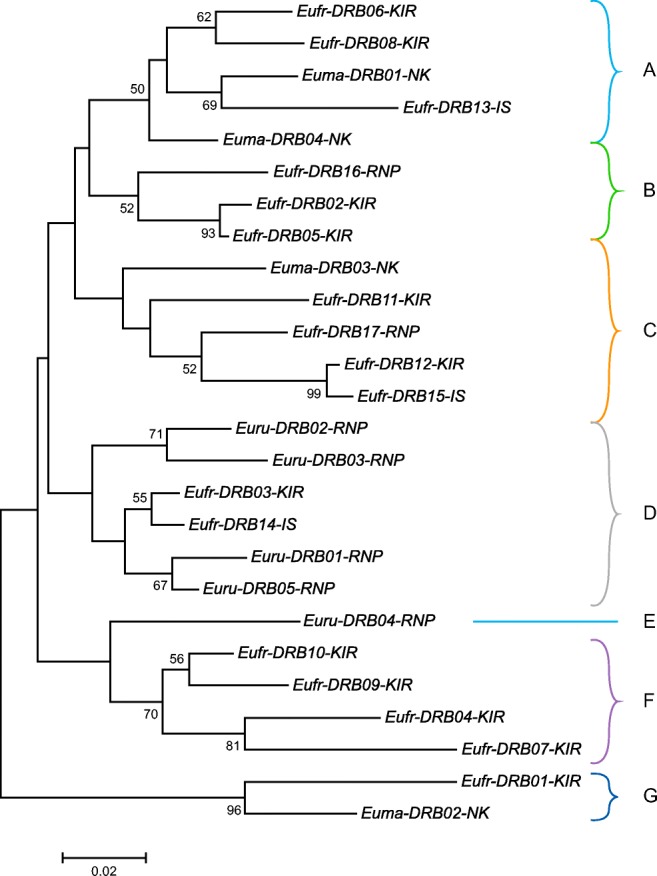
Neighbour-joining tree of exon 2 *DRB* sequences of *Eulemur macaco*, *E*. *rufifrons*, and *E*. *rubriventer*. Neighbour-joining tree constructed from 26 MHC II *DRB* exon 2 alleles in *Eulemur macaco*, *E*. *rufifrons*, and *E*. *rubriventer*. The tree was constructed in accordance with the Kimura-2-parameter model (Kimura 1980). The percentages of replicate trees in which the associated taxa cluster together in the bootstrap test are depicted in front of a node. Cluster designation is shown next to the branches (letters A–G). The tree is drawn to scale, with branch lengths in the same units as those of the evolutionary distances used. An abbreviation of the location where the *DRB* allele has been detected is given in the allele name: Nosy Komba = NK; Kirindy Forest = KIR; Ranomafana NP = RNP; and Isalo NP = IS

To visualise the phylogenetic affinities among species, we built a neighbour-joining tree, including the 26 different *DRB* alleles (Fig. 2). The branches in the resulting phylogenetic tree may indicate different *DRB* lineages (Fig. 2A–G). Each of the three species possesses one allele that clusters separately from the others, indicating evolutionarily long divergence times. The allele *Euru*-*DRB04*-*RNP*, found in *E*. *rufifrons* in Ranomafana NP, forms a single branch (Fig. 2E), whereas *Eufr*-*DRB01*-*KIR* and *Euma*-*DRB02*-*NK*, found in *E*. *rufifrons* in Kirindy Forest and *E*. *macaco* on Nosy Komba, respectively, form separate branches within one cluster (Fig. 2G). The other clusters were present in all three lemur species. Only a few *DRB* alleles within a species appear to be closely related. For example, the alleles *Eufr*-*DRB02* and *Eufr*-*DRB05* showed only two nucleotide differences and were isolated from animals from the same location, Kirindy Forest (Fig. 2B). In contrast, we identified closely related alleles with just two nucleotide differences from two different populations of the species *Eulemur rufifrons*: *Eufr*-*DRB12*-*KIR* from Kirindy Forest and *Eufr*-*DRB15*-*IS* from Isalo NP (Fig. 2C). The four alleles of *E*. *macaco* are located very distantly from each other in three different branches of the phylogenetic tree.

### Amino acid variation of the DR beta chain

**Fig. 3:**
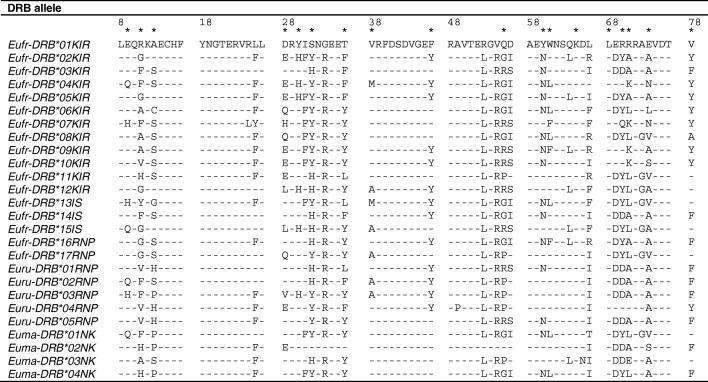
Deduced amino acid alignment of the *DRB* exon 2 of *Eulemur macaco*, *E*. *rufifrons*, and *E*. *rubriventer.* The sequence alignment starts at amino acid position 8 of exon 2. Dashes indicate identity with the first sequence. Asterisks indicate amino acids that are involved in peptide binding. Allele names are given as described for Fig. 2

All amplicons represent different *DRB* alleles, which appear to encode for unique peptides. They are composed of 71 amino acids, indicating a functional *DRB* molecule, with 28 variable amino acids (39.4%). Of the 18 ABS (Fig. 3, indicated by an asterisk), 15 sites (83.3%) are variable, while of the 53 non-ABS, only 13 (24.5%) are variable. The ratio of non-synonymous and synonymous substitution rates (dN/dS) at the complete fragments was elevated and was indicative of positive selection (*Z* = 3.99, *P* < 0.001). This can be attributed to the ABS (*Z* = 4.36, *P* < 0.001), as non-ABS did not show a significant positive selection (*Z* = 1.47, *P* = 0.07).

## Discussion

In this study, we obtained elongated *DRB* exon 2 fragments, which allowed us to define the nucleotides encoding nearly all non-synonymous codons of the antigen-binding sites. Especially amino acids 9 to 13 are of importance in encoding a peptide motif, which defines the relatedness of *DRB* alleles in humans and non-human primates (e.g., macaques), and are therefore used for lineage definition (Sommer 2005). These sequences were missing in the *DRB* amplicons of lemur *DRB* sequences published earlier in a mouse lemur species (*Microcebus murinus*) (Schad et al. 2004). Indeed, in the three lemur species analysed in this study, these amino acids were highly polymorphic, and they encode many different motifs that may be useful for phylogenetic purposes when more individuals will have been analysed (Fig. 3). Although the species in our study were all congeneric and some species lived in sympatry, alleles were neither shared between species nor between allopatric populations of a species (i.e., between the three geographically separated *E*. *rufifrons* populations)*. DRB* allele sharing of evolutionarily related species with a divergence time of less than 1.5 mya has been observed: for instance, between rhesus macaques (*Macaca mulatta*) and cynomolgus macaques (*Macaca fascicularis*) (Doxiadis et al. 2006). The absence of allele sharing among the *E*. *rufifrons* populations may indicate that these populations have been separated for more than 1.5 million years, and different parasite loads may have led to a different *DRB* repertoire. Allele sharing of the three *Eulemur* species with a far higher divergence time of ~ 4.5 mya was therefore not expected. However, as the three *E*. *rufifrons* populations are assumed to belong to the same species, it is remarkable that they do not share identical *DRB* alleles. Owing to the low sample size, however, the most plausible explanation would be that not all *DRB* alleles have yet been defined, and therefore, low-frequency shared alleles may have been missed. Furthermore, a relatively high number of *DRB* homozygous animals may indicate that due to primer inconsistencies, not all alleles have been defined. More animals need to be sampled and analysed, and calculations of observed versus expected heterozygosity are needed to evaluate whether all *DRB* alleles have been detected.

In a comparison of the different lemur species and populations, *DRB* polymorphism was largest in *E*. *rufifrons* sampled in a dry deciduous forest (Kirindy Forest). In the lemurs in this forest, the prevalence, species richness, and infection intensities of gastrointestinal parasites are high when compared to other lemur populations in Madagascar (Clough 2010). Higher and more diverse parasite loads can lead to increased individual MHC diversity (Summers et al. 2003; Harf and Sommer 2005; Clough 2010; Eizaguirre et al. 2011). Furthermore, the number of unique alleles present in the population of *E*. *macaco* on the island Nosy Komba was more than four times lower than in the *E*. *rufifrons* population in Kirindy Forest. This might be the effect of a low pathogen pressure on this island, as was also observed on the nearby island Nosy Be (Junge and Louis 2007). In addition, the low allele diversity within this isolated island population may also be the result of a small founder population, the impact of inbreeding due to the small population size, and the lack of any influx of new individuals (Frankham 2015). In contrast to various other non-human primate species, we found no evidence of duplication of the *DRB* gene in true lemurs. We cannot exclude, however, that future studies with more sensitive techniques, such as next generation sequencing, may reveal duplication(s) of the *DRB* gene. *DRB* duplication and copy number variation (CNV) of MHC genes are a common phenomenon in vertebrates and have been reported in many primate species, including chimpanzees (*Pan troglodytes*), orangutans (*Pongo pygmaeus*), macaques (Doxiadis et al. 2006), dusky titis (*Callicebus moloch*) (Trtková et al. 1993), green monkeys (*Chlorocebus sabaeus*) (Rosal-Sánchez et al. 1998), and the Senegal bushbaby (*Galago senegalensis*) (Figueroa et al. 1994). Additionally, other Strepsirrhini primates, including the northern greater galago (*Otolemur garnettii*) and the Senegal bushbaby (*Galago senegalensis*) (Figueroa et al. 1994), have a duplicated *DRB* gene. One lemur species, the grey mouse lemur (*Microcebus marinus*), also shows *DRB* duplication (Go et al. 2002; Huchard et al. 2012). However, gene duplication is rare in lemurs (Go et al. 2002; Averdam et al. 2011), and therefore, our results confirm those of previous studies, suggesting that *DRB* duplication is relatively rare in this primate group.

With the exception of two clusters, phylogenetic analysis of the *DRB* alleles of the three *Eulemur* species genotyped in this study does not show clustering of the alleles per species (Fig. 2 B, F). This may be an indication that most *DRB* alleles are older than the species’ divergence time. Instead, we saw an intermingling of alleles from different species. The long branch lengths, at least for some clusters (e.g., Fig. 2G), also indicates long evolutionary distances. As a consequence, this finding suggests that these branches represent *DRB* lineages that are shared between the three *Eulemur* species and are older than the divergence of these species. The only four alleles of *E*. *macaco* are located at a considerable distance from each other in three branches, and therefore appear to belong to different lineages. Two animals of *E*. *rufifrons* and all *E*. *rubriventer* individuals tested in this study were from Ranomafana NP, which represents a special location. It is situated on the eastern side of Madagascar and has been isolated from the rest of the island by a major mountainous geographic barrier that runs in a north-south direction over the island. The geographic separation of the populations may explain why *DRB* alleles from animals in Ranomafana NP seem less closely related to other *Eulemur DRB* alleles. The potential different external infection pressures for the separated populations may have played a role in this observation as well. However, to substantiate this suggestion, far more samples have to be analysed in the future. Additionally, when more samples become available, intron sequences should be analysed that are under less selection pressure than coding sequences in order to gain a better insight into the phylogeny of *DRB* in lemurs (Doxiadis et al. 2012).

The *DRB* genes of all three true lemur species in our study express higher rates of non-synonymous substitutions than expected under the neutrality, similar to other findings on lemurs and other primate species (reviewed in Go et al. 2002). We demonstrate a higher dN/dS ratio in the ABS compared to the dN/dS ratio of the non-ABS in the respective domains, leading to different amino acid sequences. As expected and confirmed in many other studies (Schad et al. 2004, 2005; Harf and Sommer 2005), the rate of non-synonymous substitutions did not exceed the rate of synonymous substitutions in the non-ABS. These results indicate balancing selection, leading to high levels of *DRB* diversity and polymorphism. Some studies suggest that this selection pattern is driven by parasites, for example, in grey mouse lemurs (Schad et al. 2004, 2005) and mandrills (*Mandrillus sphinx*) (Abbott et al. 2006). As the diversity in MHC II may be linked to the diversity of parasites and pathogens that can be recognised by the host (Briles et al. 1983; Langefors et al. 2001; Schad et al. 2005), the role of parasites in driving *DRB* variation needs to be investigated further.

High polymorphism levels of MHC II genes are considered critical to the long-term survival of animal populations (Edwards and Potts 1996; Grogan et al. 2017), although species with low diversity could also be viable (Sommer et al. 2002). Like all lemurs, true lemurs face significant anthropogenic threats, including disease pressures, changing climatic conditions, and habitat loss and fragmentation (Schwitzer et al. 2013; Reuter et al. 2017). Many populations have become isolated (Irwin et al. 2010), and we indicate that an isolated population in our study shows a loss of genetic diversity. Studies quantifying *DRB* alleles can assess a species’ ability to respond to the many anthropogenic threats they are facing. Especially when comparing different populations and populations with rare or unevenly distributed alleles, a greater sampling effort is needed to detect most of the *DRB* diversity. Sampling within different areas that experience anthropogenic pressures would be very interesting from a conservational perspective. We recommend conservation management to include the analysis of *DRB* polymorphism as a key to the long-term survival of endangered species, such as lemurs in Madagascar. We also recommend investigating the association between *DRB* variation and disease resistance as well as other fitness parameters in threatened populations.

## Electronic supplementary material

ESM 1 (DOCX 18 kb)
