# Supplementary material for: Determining Mhc-DRB profiles in wild populations of three congeneric true lemur species by noninvasive methods
Source: Immunogenetics. 2018 Oct 15;71(2):97–107. doi: 10.1007/s00251-018-1085-z (PMC6327083; doi:10.1007/s00251-018-1085-z)
Supplement: Supplementary file 1 — (DOCX 18 kb) [file 251_2018_1085_MOESM1_ESM.docx]

**GenBank submissions: MF682987-MF683012**

**Eulemur rufifrons MHC class II antigen (DRB) gene, DRB-Eufr-DRB*01 allele, partial cds**

GenBank: MF682987.1

[GenBank](https://www.ncbi.nlm.nih.gov/nuccore/MF682987.1?report=genbank) [Graphics](https://www.ncbi.nlm.nih.gov/nuccore/MF682987.1?report=graph) [PopSet](https://www.ncbi.nlm.nih.gov/popset?DbFrom=nuccore&Cmd=Link&LinkName=nuccore_popset&IdsFromResult=1244554044)

>MF682987.1 Eulemur rufifrons MHC class II antigen (DRB) gene, DRB-Eufr-DRB*01 allele, partial cds

CTGGAGCAGCGTAAGGCTGAGTGTCATTTCTACAACGGGACGGAGCGGGTGCGGCTCCTGGACAGATACA

TCTCTAACGGAGAAGAGACCGTGCGCTTCGACAGCGACGTGGGGGAGTTCCGGGCGGTGACGGAGCGGGG

CGTGCAGGACGCCGAGTACTGGAACAGCCAGAAGGACCTCCTGGAGCGGAGGCGGGCCGAGGTGGACACG

GTG

**Eulemur rufifrons MHC class II antigen (DRB) gene, DRB-Eufr-DRB*02 allele, partial cds**

GenBank: MF682988.1

[GenBank](https://www.ncbi.nlm.nih.gov/nuccore/MF682988.1?report=genbank) [Graphics](https://www.ncbi.nlm.nih.gov/nuccore/MF682988.1?report=graph) [PopSet](https://www.ncbi.nlm.nih.gov/popset?DbFrom=nuccore&Cmd=Link&LinkName=nuccore_popset&IdsFromResult=1244554046)

>MF682988.1 Eulemur rufifrons MHC class II antigen (DRB) gene, DRB-Eufr-DRB*02 allele, partial cds

CTGGAGCAGGGTAAGGCTGAGTGTCATTTCTACAACGGGACGGAGCGGGTGCGGTTCCTGGAGAGACACT

TCTACAACCGGGAGGAGTTCGTGCGCTTCGACAGCGACGTGGGGGAGTACCGGGCGGTGACGGAGCTGGG

CCGGGGGATCGCCGAGAACTGGAACAGCCTGAAGGACCGCCTGGATTACGCGCGGGCCGCGGTGGACACG

TAC

**Eulemur rufifrons MHC class II antigen (DRB) gene, DRB-Eufr-DRB*03 allele, partial cds**

GenBank: MF682989.1

[GenBank](https://www.ncbi.nlm.nih.gov/nuccore/MF682989.1?report=genbank) [Graphics](https://www.ncbi.nlm.nih.gov/nuccore/MF682989.1?report=graph) [PopSet](https://www.ncbi.nlm.nih.gov/popset?DbFrom=nuccore&Cmd=Link&LinkName=nuccore_popset&IdsFromResult=1244554048)

>MF682989.1 Eulemur rufifrons MHC class II antigen (DRB) gene, DRB-Eufr-DRB*03 allele, partial cds

CTGGAGCAGTTTAAGTCTGAGTGTCATTTCTACAACGGGACGGAGCGGGTGCGGCTCCTGGACAGATACA

TCCACAACCGGGAGGAGTTCGTGCGCTTCGACAGCGACGTGGGGGAGTTCCGGGCGGTGACGGAGCTGGG

CCGGCGGAGCGCCGAGAACTGGAACAGCCAGAAGGACATCCTGGATGACGCGCGGGCCGCGGTGGACACG

TTC

**Eulemur rufifrons MHC class II antigen (DRB) gene, DRB-Eufr_DRB*04 allele, partial cds**

GenBank: MF682990.1

[GenBank](https://www.ncbi.nlm.nih.gov/nuccore/MF682990.1?report=genbank) [Graphics](https://www.ncbi.nlm.nih.gov/nuccore/MF682990.1?report=graph) [PopSet](https://www.ncbi.nlm.nih.gov/popset?DbFrom=nuccore&Cmd=Link&LinkName=nuccore_popset&IdsFromResult=1244554050)

>MF682990.1 Eulemur rufifrons MHC class II antigen (DRB) gene, DRB-Eufr_DRB*04 allele, partial cds

CTGCAGCAGTTTAAGTCTGAGTGTCATTTCTACAACGGGACGGAGCGGGTGCGGTTCCTGGAGAGACACA

TCTACAACCGGGAGGAGTTCATGCGCTTCGACAGCGACGTGGGGGAGTACCGGGCGGTGACGGAGCTGGG

CCGGGGGATCGCCGAGAACTTGAACAGCCAGAAGGACCTCCTGGAGCGGAAGCGGGCTAATGTGGACACG

TAC

**Eulemur rufifrons MHC class II antigen (DRB) gene, DRB-Eufr-DRB*05 allele, partial cds**

GenBank: MF682991.1

[GenBank](https://www.ncbi.nlm.nih.gov/nuccore/MF682991.1?report=genbank) [Graphics](https://www.ncbi.nlm.nih.gov/nuccore/MF682991.1?report=graph) [PopSet](https://www.ncbi.nlm.nih.gov/popset?DbFrom=nuccore&Cmd=Link&LinkName=nuccore_popset&IdsFromResult=1244554052)

>MF682991.1 Eulemur rufifrons MHC class II antigen (DRB) gene, DRB-Eufr-DRB*05 allele, partial cds

CTGGAGCAGGGTAAGGCTGAGTGTCATTTCTACAACGGGACGGAGCGGGTGCGGTTCCTGGAGAGACACT

TCTACAACCGGGAGGAGTTCGTGCGCTTCGACAGCGACGTGGGGGAGTACCGGGCGGTGACGGAGCTGGG

CCGGGGGATCGCCGAGAACTGGAACAGCCTGAAGGACATCCTGGATTACGCGCGGGCCGCGGTGGACACG

TAC

**Eulemur rufifrons MHC class II antigen (DRB) gene, DRB-Eufr-DRB*06 allele, partial cds**

**GenBank: MF682992.1**

[GenBank](https://www.ncbi.nlm.nih.gov/nuccore/MF682992.1?report=genbank) [Graphics](https://www.ncbi.nlm.nih.gov/nuccore/MF682992.1?report=graph) [PopSet](https://www.ncbi.nlm.nih.gov/popset?DbFrom=nuccore&Cmd=Link&LinkName=nuccore_popset&IdsFromResult=1244554054)

>MF682992.1 Eulemur rufifrons MHC class II antigen (DRB) gene, DRB-Eufr-DRB*06 allele, partial cds

CTGGAGCAGGCTAAGTGTGAGTGTCATTTCTACAACGGGACGGAGCGGGTGCGGTTCCTGCAGAGATACT

TCTACAACCGGGAGGAGTACGTGCGCTTCGACAGCGACGTGGGGGAGTTCCGGGCGGTGACGGAGCTGGG

CCGGGGGATCGCCGAGAACTTGAACAGCCAGAAGGACTTCCTGGATTACTTGCGGGCCTTGGTGGACACG

TAC

# Eulemur rufifrons MHC class II antigen (DRB) gene, DRB-Eufr-DRB*07 allele, partial cds

GenBank: MF682993.1

[GenBank](https://www.ncbi.nlm.nih.gov/nuccore/MF682993.1?report=genbank) [Graphics](https://www.ncbi.nlm.nih.gov/nuccore/MF682993.1?report=graph) [PopSet](https://www.ncbi.nlm.nih.gov/popset?DbFrom=nuccore&Cmd=Link&LinkName=nuccore_popset&IdsFromResult=1244554056)

>MF682993.1 Eulemur rufifrons MHC class II antigen (DRB) gene, DRB-Eufr-DRB*07 allele, partial cds

CTGCACCAGTTTAAGTCTGAGTGTCATTTCTACAACGGGACGGAGCGGGTGCTGTACCTGCATAGATACT

TCTACAACCGGGAGGAGTACGTGCGCTTCGACAGCGACGTGGGGGAGTTCCGGGCGGTGACGGAGCTGGG

CCGGCGGAGCGCCGAGTACTTCAACAGCCAGAAGGACTTCCTGGAGCAGAAGCGGGCTAATGTGGACACG

TAC

# Eulemur rufifrons MHC class II antigen (DRB) gene, DRB-Eufr-DRB*08 allele, partial cds

GenBank: MF682994.1

[GenBank](https://www.ncbi.nlm.nih.gov/nuccore/MF682994.1?report=genbank) [Graphics](https://www.ncbi.nlm.nih.gov/nuccore/MF682994.1?report=graph) [PopSet](https://www.ncbi.nlm.nih.gov/popset?DbFrom=nuccore&Cmd=Link&LinkName=nuccore_popset&IdsFromResult=1244554058)

>MF682994.1 Eulemur rufifrons MHC class II antigen (DRB) gene, DRB-Eufr-DRB*08 allele, partial cds

CTGGAGCAGGCTAAGTCTGAGTGTCATTTCTACAACGGGACGGAGCGGGTGCGGTTCCTGCAGAGATACT

TCTACAACCGGGAGGAGTACGTGCGCTTCGACAGCGACGTGGGGGAGTTCCGGGCGGTGACGGAGCTGGG

CCGGGGGATCGCCGAGAACTTGAACAGCCAGAAGGACCGCCTGGATTACTTGCGGGGCGTGGTGGACACG

GCG

# Eulemur rufifrons MHC class II antigen (DRB) gene, DRB-Eufr-DRB*09 allele, partial cds

GenBank: MF682995.1

[GenBank](https://www.ncbi.nlm.nih.gov/nuccore/MF682995.1?report=genbank) [Graphics](https://www.ncbi.nlm.nih.gov/nuccore/MF682995.1?report=graph) [PopSet](https://www.ncbi.nlm.nih.gov/popset?DbFrom=nuccore&Cmd=Link&LinkName=nuccore_popset&IdsFromResult=1244554060)

>MF682995.1 Eulemur rufifrons MHC class II antigen (DRB) gene, DRB-Eufr-DRB*09 allele, partial cds

CTGGAGCAGGCTAAGTCTGAGTGTCATTTCTACAACGGGACGGAGCGGGTGCGGTTCCTGGAGAGATACT

TCTACAACCGGGAGGAGTACGTGCGCTTCGACAGCGACGTGGGGGAGTACCGGGCGGTGACGGAGCTGGG

CCGGCGGAGCGCCGAGAACTTCAACAGCCTGAAGGACCGCCTGGAGCGGAAGCGGGCCGCGGTGGACACG

TAC

# Eulemur rufifrons MHC class II antigen (DRB) gene, DRB-Eufr-DRB*10 allele, partial cds

GenBank: MF682996.1

[GenBank](https://www.ncbi.nlm.nih.gov/nuccore/MF682996.1?report=genbank) [Graphics](https://www.ncbi.nlm.nih.gov/nuccore/MF682996.1?report=graph) [PopSet](https://www.ncbi.nlm.nih.gov/popset?DbFrom=nuccore&Cmd=Link&LinkName=nuccore_popset&IdsFromResult=1244554062)

>MF682996.1 Eulemur rufifrons MHC class II antigen (DRB) gene, DRB-Eufr-DRB*10 allele, partial cds

CTGGAGCAGGTTAAGTCTGAGTGTCATTTCTACAACGGGACGGAGCGGGTGCGGTTCCTGGAGAGATACT

TCTACAACCGGGAGGAGTACGTGCGCTTCGACAGCGACGTGGGGGAGTACCGGGCGGTGACGGAGCTGGG

CCGGCGGAGCGCCGAGAACTGGAACAGCCAGAAGGACATCCTGGAGCGGAAGCGGGCCTCGGTGGACACG

TAC

# Eulemur rufifrons MHC class II antigen (DRB) gene, DRB-Eufr-DRB*11 allele, partial cds

GenBank: MF682997.1

[GenBank](https://www.ncbi.nlm.nih.gov/nuccore/MF682997.1?report=genbank) [Graphics](https://www.ncbi.nlm.nih.gov/nuccore/MF682997.1?report=graph) [PopSet](https://www.ncbi.nlm.nih.gov/popset?DbFrom=nuccore&Cmd=Link&LinkName=nuccore_popset&IdsFromResult=1244554064)

>MF682997.1 Eulemur rufifrons MHC class II antigen (DRB) gene, DRB-Eufr-DRB*11 allele, partial cds

CTGGAGCAGCATAAGTCTGAGTGTCATTTCTACAACGGGACGGAGCGGGTGCGGTTCCTGGAGAGATACA

TCCACAACCGGGAGGAGCTCGTGCGCTTCGACAGCGACGTGGGGGAGTTCCGGGCGGTGACGGAGCTGGG

CCGGCCGGACGCCGAGTACTGGAACAGCCAGAAGGACCGCCTGGATTACTTGCGGGGCGTGGTGGACACG

GTG

# Eulemur rufifrons MHC class II antigen (DRB) gene, DRB-Eufr-DRB*12 allele, partial cds

GenBank: MF682998.1

[GenBank](https://www.ncbi.nlm.nih.gov/nuccore/MF682998.1?report=genbank) [Graphics](https://www.ncbi.nlm.nih.gov/nuccore/MF682998.1?report=graph) [PopSet](https://www.ncbi.nlm.nih.gov/popset?DbFrom=nuccore&Cmd=Link&LinkName=nuccore_popset&IdsFromResult=1244554066)

>MF682998.1 Eulemur rufifrons MHC class II antigen (DRB) gene, DRB-Eufr-DRB*12 allele, partial cds

CTGGAGCAGGGTAAGGCTGAGTGTCATTTCTACAACGGGACGGAGCGGGTGCGGCTCCTGCTGAGACACA

TCCACAACCGGGAGGAGTACGCGCGCTTCGACAGCGACGTGGGGGAGTACCGGGCGGTGACGGAGCTGGG

CCGGCGGAGCGCCGAGTACTGGAACAGCCTGAAGGACTTCCTGGATTACTTGCGGGGCGCGGTGGACACG

GTG

# Eulemur rufifrons MHC class II antigen (DRB) gene, DRB-Eufr-DRB*13 allele, partial cds

GenBank: MF682999.1

[GenBank](https://www.ncbi.nlm.nih.gov/nuccore/MF682999.1?report=genbank) [Graphics](https://www.ncbi.nlm.nih.gov/nuccore/MF682999.1?report=graph) [PopSet](https://www.ncbi.nlm.nih.gov/popset?DbFrom=nuccore&Cmd=Link&LinkName=nuccore_popset&IdsFromResult=1244554068)

>MF682999.1 Eulemur rufifrons MHC class II antigen (DRB) gene, DRB-Eufr-DRB*13 allele, partial cds

CTGCACCAGTATAAGGGTGAGTGTCATTTCTACAACGGGACGGAGCGGGTGCGGTTCCTGGACAGATACT

TCTACAACCGGGAGGAGCTCATGCGCTTCGACAGCGACGTGGGGGAGTACCGGGCGGTGACGGAGCTGGG

CCGGGGGATCGCCGAGAACTTGAACAGCCAGAAGGACTTCCTGGATTATTTGCGGGGCGTGGTGGACACG

GTG

# Eulemur rufifrons MHC class II antigen (DRB) gene, DRB-Eufr-DRB*14 allele, partial cds

GenBank: MF683000.1

[GenBank](https://www.ncbi.nlm.nih.gov/nuccore/MF683000.1?report=genbank) [Graphics](https://www.ncbi.nlm.nih.gov/nuccore/MF683000.1?report=graph) [PopSet](https://www.ncbi.nlm.nih.gov/popset?DbFrom=nuccore&Cmd=Link&LinkName=nuccore_popset&IdsFromResult=1244554070)

>MF683000.1 Eulemur rufifrons MHC class II antigen (DRB) gene, DRB-Eufr-DRB*14 allele, partial cds

CTGGAGCAGTTTAAGTCTGAGTGTCATTTCTACAACGGGACGGAGCGGGTGCGGCTCCTGGACAGATACA

TCCACAACCGGGAGGAGTTCGTGCGCTTCGACAGCGACGTGGGGGAGTACCGGGCGGTGACGGAGCTGGG

CCGGGGGATCGCCGAGAACTGGAACAGCCAGAAGGACATCCTGGATGACGCGCGGGCCGCGGTGGACACG

TTC

# Eulemur rufifrons MHC class II antigen (DRB) gene, DRB-Eufr-DRB*15 allele, partial cds

GenBank: MF683001.1

[GenBank](https://www.ncbi.nlm.nih.gov/nuccore/MF683001.1?report=genbank) [Graphics](https://www.ncbi.nlm.nih.gov/nuccore/MF683001.1?report=graph) [PopSet](https://www.ncbi.nlm.nih.gov/popset?DbFrom=nuccore&Cmd=Link&LinkName=nuccore_popset&IdsFromResult=1244554072)

>MF683001.1 Eulemur rufifrons MHC class II antigen (DRB) gene, DRB-Eufr-DRB*15 allele, partial cds

CTGCAGCAGGGTAAGGCTGAGTGTCATTTCTACAACGGGACGGAGCGGGTGCGGCTCCTGCTGAGACACA

TCCACAACCGGGAGGAGTACGCGCGCTTCGACAGCGACGTGGGGGAGTTCCGGGCGGTGACGGAGCTGGG

CCGGCGGAGCGCCGAGTACTGGAACAGCCTGAAGGACTTCCTGGATTACTTGCGGGGCGCGGTGGACACG

GTG

# Eulemur rufifrons MHC class II antigen (DRB) gene, DRB-Eufr-DRB*16 allele, partial cds

GenBank: MF683002.1

[GenBank](https://www.ncbi.nlm.nih.gov/nuccore/MF683002.1?report=genbank) [Graphics](https://www.ncbi.nlm.nih.gov/nuccore/MF683002.1?report=graph) [PopSet](https://www.ncbi.nlm.nih.gov/popset?DbFrom=nuccore&Cmd=Link&LinkName=nuccore_popset&IdsFromResult=1244554074)

>MF683002.1 Eulemur rufifrons MHC class II antigen (DRB) gene, DRB-Eufr-DRB*16 allele, partial cds

CTGGAGCAGGGTAAGTCTGAGTGTCATTTCTACAACGGGACGGAGCGGGTGCGGTTCCTGGACAGATACA

TCCACAACCGGGAGGAGTACGTGCGCTTCGACAGCGACGTGGGGGAGTACCGGGCGGTGACGGAGCTGGG

CCGGGGGATCGCCGAGAACTTCAACAGCCTGAAGGACCGCTTGGATTACGCGCGGGCCGCGGTGGACACG

TTC

# Eulemur rufifrons MHC class II antigen (DRB) gene, DRB-Eufr-DRB*17 allele, partial cds

GenBank: MF683003.1

[GenBank](https://www.ncbi.nlm.nih.gov/nuccore/MF683003.1?report=genbank) [Graphics](https://www.ncbi.nlm.nih.gov/nuccore/MF683003.1?report=graph) [PopSet](https://www.ncbi.nlm.nih.gov/popset?DbFrom=nuccore&Cmd=Link&LinkName=nuccore_popset&IdsFromResult=1244554076)

>MF683003.1 Eulemur rufifrons MHC class II antigen (DRB) gene, DRB-Eufr-DRB*17 allele, partial cds

CTGGAGCAGGGTAAGTCTGAGTGTCATTTCTACAACGGGACGGAGCGGGTGCGGCTCCTGCAGAGATACA

TCTACAACCGGGAGGAGTACGCGCGCTTCGACAGCGACGTGGGGGAGTTCCGGGCGGTGACGGAGCTGGG

CCGGCCGGACGCCGAGTACTGGAACAGCCAGAAGGACATCCTGGATTACTTGCGGGGCGTGGTGGACACG

GTG

# Eulemur rubriventer MHC class II antigen (DRB) gene, DRB-Euru-DRB*01 allele, partial cds

GenBank: MF683004.1

[GenBank](https://www.ncbi.nlm.nih.gov/nuccore/MF683004.1?report=genbank) [Graphics](https://www.ncbi.nlm.nih.gov/nuccore/MF683004.1?report=graph) [PopSet](https://www.ncbi.nlm.nih.gov/popset?DbFrom=nuccore&Cmd=Link&LinkName=nuccore_popset&IdsFromResult=1244554078)

>MF683004.1 Eulemur rubriventer MHC class II antigen (DRB) gene, DRB-Euru-DRB*01 allele, partial cds

CTGGAGCAGGTTAAACATGAGTGTCATTTCTACAACGGGACGGAGCGGGTGCGGCTCCTGGACAGATACA

TCCACAACCGGGAGGAGTTGGTGCGCTTCGACAGCGACGTGGGGGAGTACCGGGCGGTGACGGAGCTGGG

CCGGCGGAGCGCCGAGAACTGGAACAGCCAGAAGGACATCCTGGATGACGCGCGGGCCGCGGTGGACACG

TTC

# Eulemur rubriventer MHC class II antigen (DRB) gene, DRB-Euru-DRB*02 allele, partial cds

GenBank: MF683005.1

[GenBank](https://www.ncbi.nlm.nih.gov/nuccore/MF683005.1?report=genbank) [Graphics](https://www.ncbi.nlm.nih.gov/nuccore/MF683005.1?report=graph) [PopSet](https://www.ncbi.nlm.nih.gov/popset?DbFrom=nuccore&Cmd=Link&LinkName=nuccore_popset&IdsFromResult=1244554080)

>MF683005.1 Eulemur rubriventer MHC class II antigen (DRB) gene, DRB-Euru-DRB*02 allele, partial cds

CTGCAGCAGTTTAAGTCTGAGTGTCATTTCTACAACGGGACGGAGCGGGTGCGGCTCCTGGACAGATACA

TCCACAACCGGGAGGAGTTCGCGCGCTTCGACAGCGACGTGGGGGAGTACCGGGCGGTGACGGAGCTGGG

CCGGCCGGACGCCGAGTACTGGAACAGCCAGAAGGACATCCTGGATGACGCGCGGGCCGCGGTGGACACG

TTC

# Eulemur rubriventer MHC class II antigen (DRB) gene, DRB-Euru-DRB*03 allele, partial cds

GenBank: MF683006.1

[GenBank](https://www.ncbi.nlm.nih.gov/nuccore/MF683006.1?report=genbank) [Graphics](https://www.ncbi.nlm.nih.gov/nuccore/MF683006.1?report=graph) [PopSet](https://www.ncbi.nlm.nih.gov/popset?DbFrom=nuccore&Cmd=Link&LinkName=nuccore_popset&IdsFromResult=1244554082)

>MF683006.1 Eulemur rubriventer MHC class II antigen (DRB) gene, DRB-Euru-DRB*03 allele, partial cds

CTGCACCAGTTTAAGCCTGAGTGTCATTTCTACAACGGGACGGAGCGGGTGCGGTTCCTGGTGAGACACA

TCTACAACCGGGAGGAGTACGCGCGCTTCGACAGCGACGTGGGGGAGTACCGGGCGGTGACGGAGCTGGG

CCGGCCGGACGCCGAGTACTGGAACAGCCAGAAGGACATCCTGGATGACGCGCGGGCCGCGGTGGACACG

TTC

# Eulemur rubriventer MHC class II antigen (DRB) gene, DRB-Euru-DRB*04 allele, partial cds

GenBank: MF683007.1

[GenBank](https://www.ncbi.nlm.nih.gov/nuccore/MF683007.1?report=genbank) [Graphics](https://www.ncbi.nlm.nih.gov/nuccore/MF683007.1?report=graph) [PopSet](https://www.ncbi.nlm.nih.gov/popset?DbFrom=nuccore&Cmd=Link&LinkName=nuccore_popset&IdsFromResult=1244554084)

>MF683007.1 Eulemur rubriventer MHC class II antigen (DRB) gene, DRB-Euru-DRB*04 allele, partial cds

CTGGAGCAGGTTAAACATGAGTGTCATTTCTACAACGGGACGGAGCGGGTGCGGTTCCTGGAGAGATACA

TCTACAACCGGGAGGAGTTCGTGCGCTTCGACAGCGACGTGGGGGAGTACCGGCCGGTGACGGAGCTGGG

CCGGCCGGACGCCGAGTACTGGAACAGCCAGAAGGACATCCTGGAGCGGAGGCGGGCCGCGGTGGACACG

TAC

# Eulemur rubriventer MHC class II antigen (DRB) gene, DRB-Euru-DRB*05 allele, partial cds

GenBank: MF683008.1

[GenBank](https://www.ncbi.nlm.nih.gov/nuccore/MF683008.1?report=genbank) [Graphics](https://www.ncbi.nlm.nih.gov/nuccore/MF683008.1?report=graph) [PopSet](https://www.ncbi.nlm.nih.gov/popset?DbFrom=nuccore&Cmd=Link&LinkName=nuccore_popset&IdsFromResult=1244554086)

>MF683008.1 Eulemur rubriventer MHC class II antigen (DRB) gene, DRB-Euru-DRB*05 allele, partial cds

CTGGAGCAGGTTAAACATGAGTGTCATTTCTACAACGGGACGGAGCGGGTGCGGTTCCTGGACAGATACA

TCTACAACCGGGAGGAGTACGTGCGCTTCGACAGCGACGTGGGGGAGTTCCGGGCGGTGACGGAGCTGGG

CCGGCGGAGCGCCGAGAACTGGAACAGCCAGAAGGACATCCTGGATGACGCGCGGGCCGCGGTGGACACG

TTC

# Eulemur macaco MHC class II antigen (DRB) gene, DRB-Euma-DRB*01 allele, partial cds

GenBank: MF683009.1

[GenBank](https://www.ncbi.nlm.nih.gov/nuccore/MF683009.1?report=genbank) [Graphics](https://www.ncbi.nlm.nih.gov/nuccore/MF683009.1?report=graph) [PopSet](https://www.ncbi.nlm.nih.gov/popset?DbFrom=nuccore&Cmd=Link&LinkName=nuccore_popset&IdsFromResult=1244554088)

>MF683009.1 Eulemur macaco MHC class II antigen (DRB) gene, DRB-Euma-DRB*01 allele, partial cds

CTGCAGCAGTTTAAGCCTGAGTGTCATTTCTACAACGGGACGGAGCGGGTGCGGCTCCTGGACAGATACT

TCTACAACCGGGAGGAGTACGTGCGCTTCGACAGCGACGTGGGGGAGTTCCGGGCGGTGACGGAGCTGGG

CCGGGGGATCGCCGAGAACTTGAACAGCCAGAAGGACACCCTGGATTACTTGCGGGGCGTGGTGGACACG

GTG

# Eulemur macaco MHC class II antigen (DRB) gene, DRB-Euma-DRB*02 allele, partial cds

GenBank: MF683010.1

[GenBank](https://www.ncbi.nlm.nih.gov/nuccore/MF683010.1?report=genbank) [Graphics](https://www.ncbi.nlm.nih.gov/nuccore/MF683010.1?report=graph) [PopSet](https://www.ncbi.nlm.nih.gov/popset?DbFrom=nuccore&Cmd=Link&LinkName=nuccore_popset&IdsFromResult=1244554090)

>MF683010.1 Eulemur macaco MHC class II antigen (DRB) gene, DRB-Euma-DRB*02 allele, partial cds

CTGGAGCAGCATAAGCCTGAGTGTCATTTCTACAACGGGACGGAGCGGGTGCGGTTCCTGGAGAGATACA

TCTCTAACGGAGAAGAGACCGTGCGCTTCGACAGCGACGTGGGGGAGTTCCGGGCGGTGACGGAGCGGGG

CGTGCAGGACGCCGAGTACTGGAACAGCCAGAAGGACATCCTGGATGACGCGCGGGCCTCGGTGGACACG

TTC

# Eulemur macaco MHC class II antigen (DRB) gene, DRB-Euma-DRB*03 allele, partial cds

GenBank: MF683011.1

[GenBank](https://www.ncbi.nlm.nih.gov/nuccore/MF683011.1?report=genbank) [Graphics](https://www.ncbi.nlm.nih.gov/nuccore/MF683011.1?report=graph) [PopSet](https://www.ncbi.nlm.nih.gov/popset?DbFrom=nuccore&Cmd=Link&LinkName=nuccore_popset&IdsFromResult=1244554092)

>MF683011.1 Eulemur macaco MHC class II antigen (DRB) gene, DRB-Euma-DRB*03 allele, partial cds

CTGGAGCAGGCTAAGTCTGAGTGTCATTTCTACAACGGGACGGAGCGGGTGCGGTTCCTGGACAGATACA

TCCACAACCGGGAGGAGTACGTGCGCTTCGACAGCGACGTGGGGGAGTTCCGGGCGGTGACGGAGCTGGG

CCGGCCGGACGCCGAGTACTGGAACAGCCTGAAGAACATCCTGGATGACGAGCGGGCCGCGGTGGACACG

GTG

# Eulemur macaco MHC class II antigen (DRB) gene, DRB-Euma-DRB*04 allele, partial cds

GenBank: MF683012.1

[GenBank](https://www.ncbi.nlm.nih.gov/nuccore/MF683012.1?report=genbank) [Graphics](https://www.ncbi.nlm.nih.gov/nuccore/MF683012.1?report=graph) [PopSet](https://www.ncbi.nlm.nih.gov/popset?DbFrom=nuccore&Cmd=Link&LinkName=nuccore_popset&IdsFromResult=1244554094)

>MF683012.1 Eulemur macaco MHC class II antigen (DRB) gene, DRB-Euma-DRB*04 allele, partial cds

CTGGAGCAGCATAAGCCTGAGTGTCATTTCTACAACGGGACGGAGCGGGTGCGGTTCCTGGACAGATACT

TCTACAACCGGGAGGAGTACGTGCGCTTCGACAGCGACGTGGGGGAGTTCCGGGCGGTGACGGAGCTGGG

CCGGGGGATCGCCGAGAACTTGAACAGCCAGAAGGACATCCTGGATTACTTGCGGGCCGCGGTGGACACG

TTC
